# Can saliva offer an advantage in monitoring of diabetes mellitus? – A case control study

**DOI:** 10.4317/jced.51386

**Published:** 2014-10-01

**Authors:** Preethi Balan, Subhas G. Babu, Kumari N. Sucheta, Shishir R. Shetty, Anusha L. Rangare, Renita L. Castelino, Areekat K. Fazil

**Affiliations:** 1 MDS, Senior lecturer. Department of Oral medicine and Radiology. Sree Anjaneya institute of dental sciences. Nitte University, Mangalore; 2 MDS. Department of Oral medicine and Radiology. AB Shetty Memorial Institute of Dental Sciences. Nitte University, Mangalore; 3 MD Biochemistry. K.S. Hegde Medical Academy. Nitte University, Mangalore; 4 MDS. Department of Oral medicine and Radiology. Century International Institute of dental Sciences & Research Center. KUHS University, Kasargode

## Abstract

Objectives: Diabetes Mellitus is emerging as a major health problem over these years. Present method of blood glucose monitoring by venepuncture is invasive leading to reduced patient compliance and thereby ineffective judicious monitoring. The need of the hour is to direct research in the direction of establishing painless and more acceptable blood glucose analysis method.The objective of the study is to conduct a comparative analysis of the concentrations of salivary glucose and blood glucose in type 2 diabetes mellitus patients. Study Design: This study assessed glucose levels using the glucose oxidase method in blood and unstimulated saliva in 90 subjects who were divided into 3 equal groups of controlled type 2 diabetes, uncontrolled type 2 diabetes and those without diabetes. Statistical analysis was carried out using one way ANOVA, Post hoc Tukeys tests and Pearson’s correlation coefficient test. Results: Salivary glucose levels were significantly higher in patients with diabetes than controls. There was a significant positive correlation between salivary and plasma glucose levels in patients with diabetes. Conclusions: Glucose concentration in saliva is higher in diabetics but hyperglycemia does not influence salivary glucose levels. Further clarification is required to claim the diagnostic potentials of saliva in diabetes.

** Key words:**Salivary glucose, type 2 diabetes mellitus, blood glucose.

## Introduction

Diabetes mellitus is a complex multisystem disorder (1) with a rising global prevalence of 6.4% in adult popula-tion and worldwide (2,3). It is emerging as a major health problem because of the high morbidity and mortality associated with infections and renal, retinal and vascular complications (4). The two key aspects of diabetic management are normalization of blood glucose level and its judicious monitoring; both of these need patient’s regular compliance. Currently, blood glucose estimation is carried out by venepuncture, which causes physical and psychological trauma to patient rendering them more apprehensive about monitoring diabetic status. The need of the hour is to direct research in the direction of establishing painless and more acceptable blood glucose analysis methods which will not hamper the regular visit of patients to diabetic clinic. Saliva is an organic fluid that is easy to collect by non-invasive methods and is not costly to preserve (5). The salivary glucose level has been reported to closely reflect blood glucose level prompting it to be used as a diagnostic resource as it offers a distinctive advantage of being a noninvasive procedure allowing multiple sampling.

Considering the increased incidence of diabetes mellitus worldwide, limited amount of studies carried out, we proposed to conduct a comparative analysis of the concentrations of salivary glucose and blood glucose in type 2 diabetes mellitus patients.

## Material and Methods

The present study is a randomized case control study conducted in the Department of Oral Medicine and Maxillofacial Radiology, of a dental college in south India during 2011-2012. The study was approved by the ethics committees of the University. Participants provided written informed consent prior to data collection.

- Study population

The study sample consisted of 90 subjects divided into 3 equal groups of 30 patients each of either gender, between the age of 30 years to 60 years. Briefly, Group I comprised of control patients with Random Non Fasting Plasma Glucose (RNFPG) levels less than 120 mg/dl; Group II included patients with controlled diabetes mellitus (RNFPG levels in the range of 120 mg/dl to 200 mg/dl); Group III consisted of patients with uncontrolled diabetes mellitus (RNFPG levels more than 200 mg/dl). Excluded from the study were patients with history of any systemic or oral mucosal disease, patients who are on medications other than anti-diabetic drugs and patients with habits like smoking, tobacco or betel nut chewing and alcohol consumption. The most recent value of HbA1c, not exceeding 3 months, was obtained from the patient’s medical chart to assess the diabetic status of the patient.

- Blood sampling

Employing aseptic precautions 2 ml peripheral venous blood was collected from the antecubital vein with syringe into a test-tube containing 20µl fluoride oxalate to prevent clotting of blood and degradation of glucose. The blood glucose was measured using glucose oxidase method employing an autoanalyser.

- Saliva Collection

Patients were instructed to abstain from eating for 2 hours before the sample collection. Unstimulated saliva was collected using a “spit technique”. The patient was asked to sit in the erect with head tilted forward and instructed not to speak, swallow, or do any head movements during the procedure, or swallow any saliva if present in the mouth. Then the patient was instructed to spit in a sterile graduated container every minute for 10 minutes.

Salivary samples thus collected represented whole mouth fluid contributed by secretions from major and minor salivary glands and potentially gingival crevicular fluid.

- Salivary glucose estimation

Salivary glucose level was determined by using glucose oxidase-peroxidase (GOD-POD) method. In this method, initial enzymatic oxidation of glucose by glucose oxidase (GOD) enzyme takes place. The colorimetric indicator is quinone, which is generated from 4 aminoantipyrine and phenol by hydrogen peroxide under the catalytic action of peroxidase (POD) (Trinder’s reaction).

A pipette was used to transfer 1000 ?l of reagent solution into 3 test tubes each labeled as ‘Blank’, ‘Standard’ and ‘Test’. Then, 10 ?l of standard was added to the test tube marked as ‘Standard’, followed by 10 ?l of test sample to the ‘Test’ test tube. The sample was mixed and incubated for 10 minutes at 37o C. Absorbance was measured within 60 minutes at 505 nm against reagent blank using Roche automated clinical chemistry analyzer.

- Statistical study

The information was organized and analyzed with the help of Statistical Package for the Social Sciences (SPSS) version 18 for Windows. All values were expressed as means ± standard deviation (SD) and *P* (probability) values of < 0.001 were considered to be significant. The Analysis of Variance (ANOVA) and Post hoc Tukeys tests were employed for comparison between the groups and for comparison of the 2 study groups with control groups respectively.

Pearson’s correlation coefficient test was applied to assess the correlation between blood glucose level and salivary glucose level.

## Results

The mean salivary glucose level in group I was calculated to be 1.18 ± 0.675 mg/dl which was lower than the mean salivary glucose level in group II which was measured 4.95 ± 2.479 mg/dl. The mean salivary glucose level in group III was 13.35 ± 6.61 mg/dl which was highest ( Table 1). The mean serum glucose level was highest in group III followed by group II and Group I ( Table 2). Statistically significant difference was obtained when comparison of salivary glucose levels between study groups was done (*P* < 0.001), while the comparison of control group with group II revealed no significant difference (*P* < 0.002) ( Table 3).

**Table 1:**
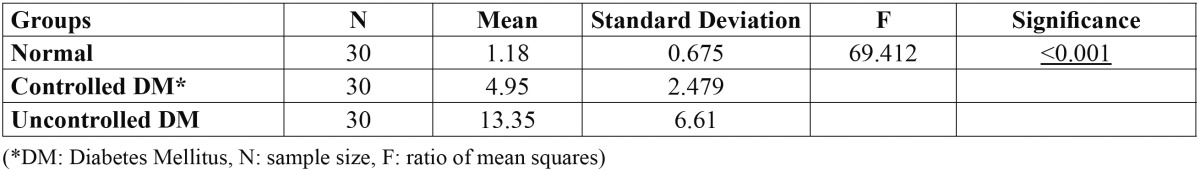
Comparison of study and control groups with respect to salivary glucose using one way ANOVA.

**Table 2:**
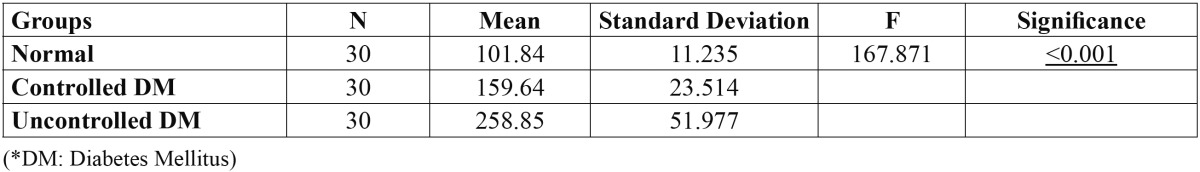
Comparison of study and control groups with respect to blood glucose using one way ANOVA.

**Table 3:**
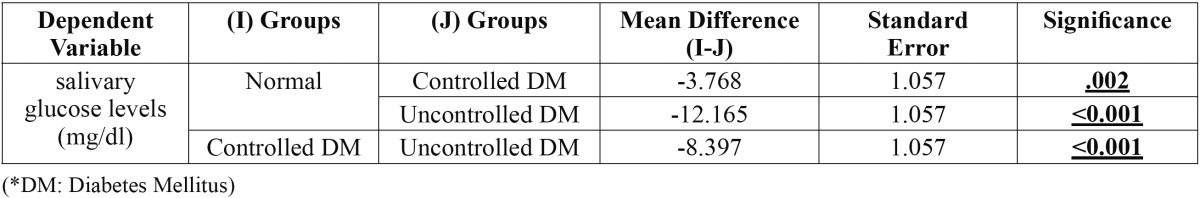
Analysis of statistical significance of salivary glucose levels using post hoc tukeys test.

The correlation between blood and salivary glucose levels was found to be excellent between the study groups with 80.2816 % cases of group II showing the correlation (r value = 0.896) and 74.1321 % cases of group III showing the correlation (r value = 0.861) ( Table 4).

**Table 4:**
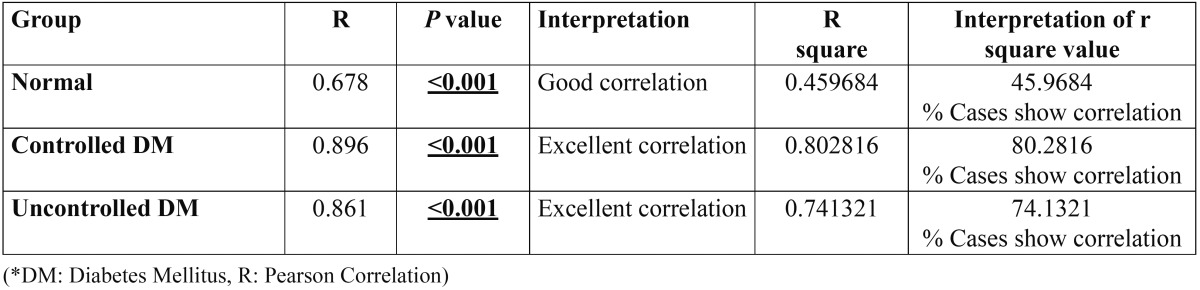
Correlation between salivary and blood glucose level in study groups and control group.

## Discussion

The present study was conducted using saliva as a diagnostic fluid which is becoming increasingly apparent to investigators and clinician in large scale screening and epidemiological studies.

In the present study, glucose was detected in the saliva of nondiabetic and diabetic subjects, which was in confirmatory with studies by Darwazeh *et al.* (6) and Ben-Aryeh *et al.* (7). However, salivary glucose levels were significantly higher in uncontrolled diabetic subjects (13.35 ± 6.61 mg/dl) and controlled diabetic subjects (4.95 ± 2.479 mg/dl) than in nondiabetic subjects (1.18 ± 0.675 mg/dl) which was in accordance with previous studies by Lasisi *et al.* (8), Carda et al. (9), Aydin (10) and Thorstensson *et al.* (11). Salivary glucose levels are likely to follow a threshold mechanism. This increase in salivary glucose levels with increase in blood glucose levels has been attributed to “leakage” across the basement membrane of the glands, particularly the parotid gland, when blood glucose levels increase beyond a threshold value (12). The elevated salivary glucose level in diabetes also confirms the effects of diabetic membranopathy, which leads to raised percolation of glucose from blood to saliva thus altering the salivary composition in diabetes mellitus (13).

However, studies by Sharon *et al.* (14) and Andersson *et al.* (15) showed elevated salivary glucose levels only in parotid saliva, while Marchetti *et al.* (16) reported no changes in salivary glucose levels in patient with diabetes. This detection limit could also reflect the sensitivity of the test used. Salivary samples collected in the present study represent whole-mouth fluid and therefore reflect glucose levels not only due to leakage across basement membrane of major and minor salivary glands but potentially also from gingival crevicular fluid (14). The increase in salivary glucose can be attributed to the carbohydrate-rich dietary pattern of the Indian population which had been the focus group of this study. Investigative studies if carried out in future to analyze the glucose concentration in various oral secretions after segregation can contribute to accuracy of salivary glucose estimation and help solve the controversies revolving around the detection of salivary glucose.

Statistically significant difference was obtained when comparison of salivary glucose levels between group of patients with controlled and uncontrolled diabetes was carried out (*P* < 0.001). The results were not significant on comparison between control group and group with patients with controlled diabetes (*P* = 0.002). According to Lopez *et al.* (17) the salivary glands act as filters of blood glucose that are altered by hormonal or neural regulation. Persistent hyperglycemia leads to microvascular changes in the blood vessels, as well as basement membrane alteration in the salivary glands. This leads to increased leakage of glucose from the ductal cells of the salivary gland, thereby increasing the glucose content in saliva (18). Thus salivary glucose is not directly influenced by glycemia and its level is affected by confounding factors like degree of metabolic control and extent of salivary gland damage suggesting a doubtful linear relationship between blood and salivary glucose.

In the present study, the correlation between blood and salivary glucose levels was found to be excellent with 80.2816 % patients with controlled diabetes (*P* < 0.00, r = 0.896) and 74.1321% patients with uncontrolled diabetes (*P* < 0.001, r = 0.861) showing the correlation. Comparatively, the correlation observed between blood and salivary glucose levels in patients without diabetes was not strong with 45.9684 % of patients (*P* < 0.001, r = 0.678) showing the correlation. Abikshyeet *et al.* (18) also observed a positive correlation between salivary and serum glucose in diabetic patients and controls as well and these correlations were found to be statistically significant. In disagreement with report of ours, Vasconcelos *et al.* (19) did not observe correlation between the level of capillary blood glycemia and concentration of salivary glucose.

The observations derived from this study require more comprehensive evaluation with emphasis on saliva sampling methods as disparities in results have been noticed in the research work carried out so far because raised glucose level varies according to the type of salivary samples used - whole saliva or saliva collected from the individual salivary glands. The role of salivary gland status in influencing salivary glucose level also needs evaluation as the quantity of leakage of glucose from plasma to saliva depends more on the amount of damage to the salivary gland due to hyperglycemia which is unpredictable. The variation seen in the salivary glucose levels in the various studies carried out so far may be due to the focus on different population groups with diverse dietary pattern. Thus, evaluation of the relation of population selected with salivary glucose levels can help standardizing the results.

Nevertheless, the present study may give a new perspective for further investigations on the regulation of glucose output from salivary glands, as well as on the diagnostic potentials of saliva as a non invasive means for monitoring glycaemia.
